# Alk1/Endoglin signaling restricts vein cell size increases in response to hemodynamic cues

**DOI:** 10.1007/s10456-024-09955-3

**Published:** 2024-12-10

**Authors:** Zeenat Diwan, Jia Kang, Emma Tsztoo, Arndt F. Siekmann

**Affiliations:** 1 Department of Cell and Developmental Biology, Perelman School of Medicine at the University of Pennsylvania, 1114 Biomedical Research Building, 421 Curie Boulevard, Philadelphia, PA 19104 USA; 2 Department of Bioengineering, School of Engineering and Applied Science, University of Pennsylvania, Philadelphia, PA USA

**Keywords:** Endoglin, Alk1, Hereditary hemorrhagic telangiectasia, Shear Stress Set Point, Endothelial Cell Size, Artery, Vein, Zebrafish

## Abstract

Hemodynamic cues are thought to control blood vessel hierarchy through a shear stress set point, where flow increases lead to blood vessel diameter expansion, while decreases in blood flow cause blood vessel narrowing. Aberrations in blood vessel diameter control can cause congenital arteriovenous malformations (AVMs). We show in zebrafish embryos that while arteries behave according to the shear stress set point model, veins do not. This behavior is dependent on distinct arterial and venous endothelial cell (EC) shapes and sizes. We show that arterial ECs enlarge more strongly when experiencing higher flow, as compared to vein cells. Through the generation of chimeric embryos, we discover that this behavior of vein cells depends on the bone morphogenetic protein (BMP) pathway components Endoglin and Alk1. *Endoglin* (*eng*) or *alk1* (*acvrl1*) mutant vein cells enlarge when in normal hemodynamic environments, while we do not observe a phenotype in either *acvrl1* or *eng* mutant ECs in arteries. We further show that an increase in vein diameters initiates AVMs in *eng* mutants, secondarily leading to higher flow to arteries. These enlarge in response to higher flow through increasing arterial EC sizes, fueling the AVM. This study thus reveals a mechanism through which BMP signaling limits vein EC size increases in response to flow and provides a framework for our understanding of how a small number of mutant vein cells via flow-mediated secondary effects on wildtype arterial ECs can precipitate larger AVMs in disease conditions, such as hereditary hemorrhagic telangiectasia (HHT).

**Supplementary Information:**

The online version contains supplementary material available at 10.1007/s10456-024-09955-3.

## Introduction

Tubular networks are the fundamental structural units of many organs, including the vascular system [1]. A hierarchical organization of correctly sized tubes ensures an appropriate distribution of liquids or air to all parts of the body. Aberrations in the diameter of these vessels can disrupt this hierarchy and cause congenital vascular disorders, including Hereditary Hemorrhagic Telangiectasia (HHT), Cerebral Cavernous Malformations (CCM) and Venous and Lymphatic Malformations [2]. Understanding the mechanisms regulating blood vessel diameters in vivo will therefore not only shed light on the processes shaping biological tubes, but also help in developing new therapies for vascular disorders.

Feedback mechanisms exist between the forces (fluid shear stress or FSS among them) that the flowing blood exerts on a given blood vessel and its diameter [3, 4]. The shear stress set point theory proposes that FSS below or above a specific threshold initiates vessel remodelling: constricting for low FSS and dilating for high FSS, to return to the original shear stress level and uphold homeostasis [5, 6]. However, the existence of a shear stress set point for both arteries and veins would mean that the unchecked increase in blood flow during embryonic development would result in continual increases in vessel diameters of the shortest connection between an artery and a vein, leading to the formation of an arteriovenous shunt [7]. Therefore, mechanisms must exist to counteract the effects of shear stress set point-driven blood vessel dilations. Mural cells are known to regulate blood vessel dilation and constriction in response to shear stress [8]. However, mural cells do not mature until later in development, and in the zebrafish axial vasculature this occurs at around 72 h post fertilization (hpf) [9]. Hence, during earlier developmental stages, blood vessel diameter control relies on endothelial cell (EC) specific mechanisms. While it is well established that gene expression differences exist between ECs of distinct blood vessel types, few studies have analysed how these differences might affect EC morphologies [10, 11]. It is also not clear, whether differences in blood vessel diameters are due to differences in EC numbers or shapes [12, 13].

The analysis of animal models that develop arteriovenous shunts has provided inroads into these mechanisms. A loss of function of BMP pathway components, such as Endoglin, Alk1 or Smad4, frequently results in arteriovenous malformations (AVMs), both in human patients suffering from HHT and in zebrafish and mouse models of the disease [14–20]. These studies suggested that either increases in EC numbers (through proliferation or migration) or changes in EC sizes are the main driver to increase vessel diameters. A recent study investigating SMAD4 loss of function in ECs further showed that mutant cells had increased sensitivity to flow, ultimately leading to loss of arterial identity because of excessive EC proliferation [21]. Blocking proliferation can rescue AVM formation in mouse models of HHT [21–23]. Loss of arterial marker gene expression, such as *Efnb2* and *Jag1* in *acvrl1* (*alk1*) mutant mice in addition to tracking of AVM onset in *eng* mutant mouse retinae suggested that arteries or arterioles are the first vessel segments displaying a phenotype during AVM development [24–26]. Arterial phenotypes were also reported for *alk1* mutant zebrafish [19] and for loss of SMAD4 during the development of the vasculature in the mouse heart, which specifically affected coronary arterial ECs [27]. Other studies in the mouse retina, however, showed that Alk1, Endoglin and SMAD4 were required in venous and capillary ECs while being dispensable in arteries [28–30]. Early observations of vascular lesions in HHT patients reported that postcapillary venule sizes were the first to increase, like studies in mice [31, 32]. Together, these results suggest that it has either been difficult to distinguish primary from secondary effects caused by loss of Alk1/Endoglin signaling or that distinct mechanisms trigger arteriovenous malformations depending on the tissue and/or vascular bed analyzed.

Here, we report that the dorsal aorta and the posterior cardinal vein of zebrafish embryos consist of ECs of different shapes and sizes and that flow increases occurring during normal embryonic development result in an increase in arterial EC sizes, while vein ECs despite also experiencing flow increases do not enlarge. Alk1/Endoglin signaling is required within ECs of the posterior cardinal vein to restrict their size increase, while being dispensable within arterial ECs. Of importance, we previously showed that ECs in the dorsal aorta enlarged in *eng* mutant zebrafish embryos [20]. Our current study indicates that this expansion is likely a secondary consequence due to increases in blood flow. Together, these results link the development of appropriate vascular patterns in response to changing hemodynamic cues with distinct morphological responses of venous and arterial ECs to these cues. Additionally, they emphasize the significance of Alk1/Endoglin signalling in a cell-type-specific manner throughout this process.

## Results

### The dorsal aorta and posterior cardinal vein show differential growth during development and are made up of ECs with distinct dimensions

**Fig. 1:**
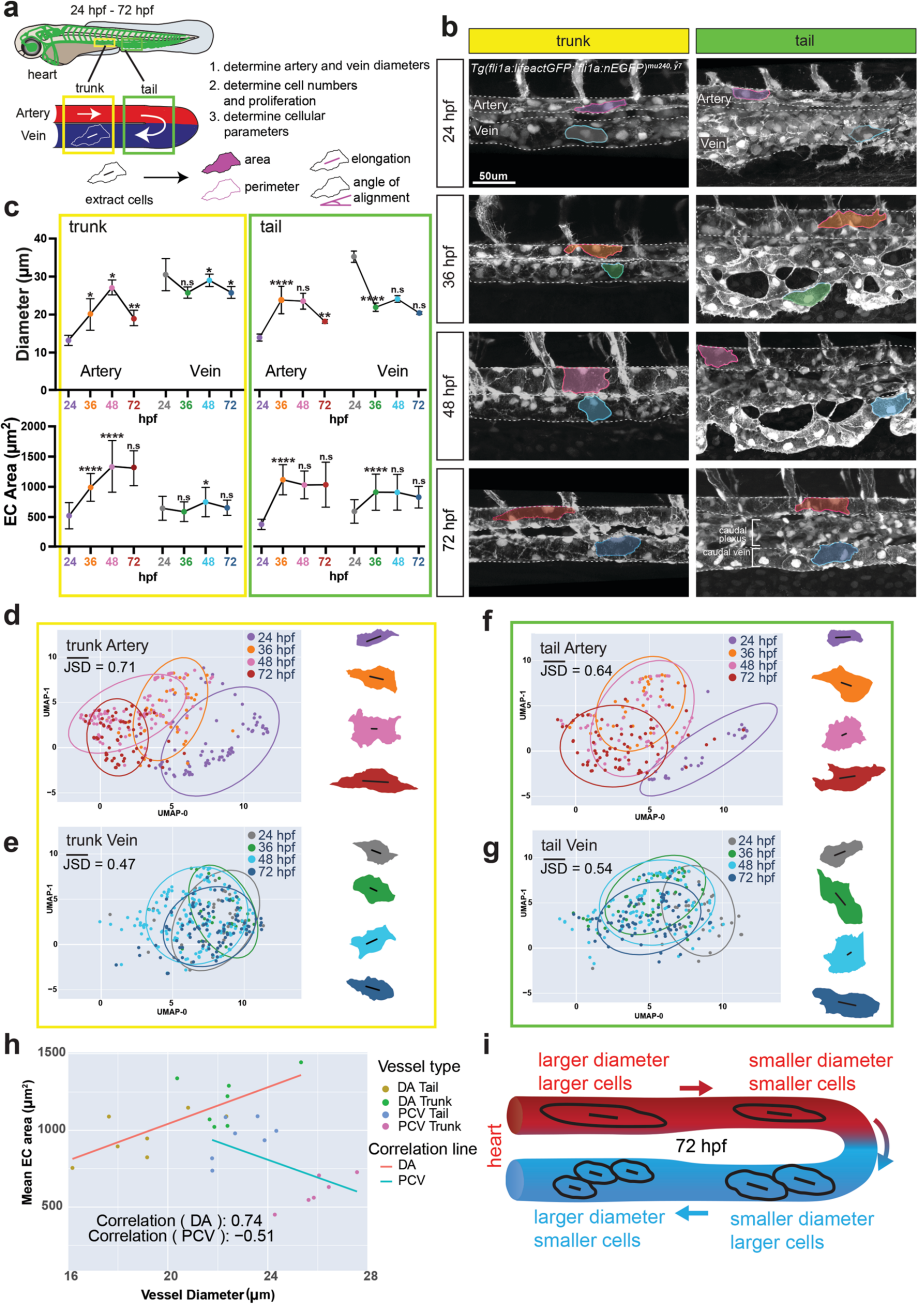
The dorsal aorta and posterior cardinal vein show differential growth during development. **a** Schematic of a wildtype zebrafish embryo, vasculature highlighted in green. The outlined yellow and green boxes indicate the trunk and tail regions, respectively, with magnified views underneath. White arrows represent blood flow. Numbered bullets highlight step-by-step process of morphometric analysis with a schematic of cellular parameters analysed. **b** Timelapse images of the dorsal aorta and posterior cardinal vein in the trunk and tail regions of wildtype embryos at 24, 36, 48 and 72 hpf. Representative ECs outlined to show differences and changes in EC morphology. n = 3 movies. **c** Quantifications of vessel diameter and EC area of artery and vein in the trunk and tail regions. Data analysed across developmental stages by two-way ANOVA. n.s, not significant; *P < 0.05, **P < 0.01, ***P < 0.001, ****P < 0.0001; error bars indicate s.d. *n* = 33–77 cells from trunk artery, 27–71 cells from trunk vein, 21–39 cells from tail artery, 30–63 cells from tail vein, from at least 3 wildtype embryos at 24, 36, 48 and 72 hpf each. **d–g** UMAP plots and representative 2D cell outlines of arterial and venous ECs at 24, 36, 48 and 72 hpf in the trunk (**d, e**) and tail (**f, g**) regions. Length and angle of black line within each cell represents elongation and angle of alignment of the cell, respectively. JSD-Jensen Shannon Divergence. **h** Correlation graph plotting the coefficient of correlation between Mean EC area and Vessel diameter of DA and PCV ECs in the trunk and tail of 72 hpf embryos. **i** Schematic of vessel diameters and EC morphologies in the artery and vein of wildtype embryos at 72 hpf

To investigate the mechanisms regulating blood vessel sizes during embryonic development, we performed timelapse imaging of the major artery (dorsal aorta, DA) and major vein (posterior cardinal vein, PCV) in the zebrafish embryo starting at 24 hpf (when the vessels have just formed and lumenized) until 72 hpf (Fig. 1a). We further distinguished between anterior (trunk) and posterior (tail) DA and PCV regions, since they experience differences in blood flow magnitude [33]. To determine whether blood vessel diameters depend on differences in cell numbers or cell shapes, we measured DA and PCV diameters and determined EC shapes and numbers. We observed that in the trunk region, the vein started out with a significantly larger diameter than the artery at 24 hpf (Fig. 1b, c). Arterial diameters increased from 24 to 48 hpf, correlating with increases in blood flow magnitude [34]. By contrast, the vein showed minimal fluctuations in diameter during this time interval. Using *Tg(fli1a:nEGFP)*^*y7*^ embryos to label EC nuclei, we discovered that the vein had significantly higher EC numbers compared to the artery (Suppl. Figure 1a), while venous EC proliferation rate was not significantly different from that of the artery (Suppl. Figure 1b, i, j). Both arterial and venous EC numbers decreased over time (Suppl. Figure 1a), correlating with migration of ECs out of the vessels [35]. Thus, the observed increases in arterial diameters between 24 and 48 hpf cannot be explained by increases in EC numbers. However, while DA and PCV EC areas were similar at 24 hpf, at the onset of blood flow, arterial ECs subsequently grew in area to accomodate DA diameter increases (Fig. 1c, Artery). By contrast, the change in PCV diameters and EC areas was much smaller during this time window (Fig. 1c, Vein). Nevertheless, the alignment angle for both arterial and venous ECs decreased between 48 and 72 hpf (Suppl. Figure 1c), correlating with a decrease in vessel diameter (Fig. 1c). In conclusion, our observations indicate that the trunk artery grows in diameter through an increase in EC sizes between 24 and 48 hpf, while the trunk vein undergoes marginal changes, both in terms of diameter and EC areas over time.

We made similar observations concerning the tail vessels, despite the tail vein undergoing extensive angiogenic remodelling between 24 and 48 hpf to give rise to two distinct but connected structures—the caudal plexus and the caudal vein (Fig. 1b, see tail images) [36]. This splitting of the vessel led to a significant decrease in tail vein diameter between 24 and 36 hpf (Fig. 1c). Of note, the differences between tail artery and vein diameters and EC sizes were less pronounced when compared to trunk vessels (Fig. 1b, c; Suppl. Figure 1e–h). Like tail artery ECs, and different from trunk vein ECs, tail vein ECs also increased in size over time. To obtain a more comprehensive view of differences in cell shapes between arteries and veins and how they change over time, we displayed all EC shape parameters (area, perimeter, elongation, and angle) in Uniform Manifold Approximation and Projection (UMAP) plots (Fig. 1d–g). This analysis revealed that vein cells differed less over time when compared to arterial ECs, especially in the trunk region (Fig. 1d, e). We chose the statistical analysis Jensen-Shannon Divergence (JSD) to measure the divergence of cell shape distribution between two cell types in their original 4-dimensional space. The value of JSD is bounded between 0 and 1. The similarity between the distributions is greater when the Jensen-Shannon distance is closer to zero, with 0 meaning that they are identical.Together, these findings show a remarkable diversity in EC shapes not only between arterial and venous blood vessels, but also dependent on their anterior–posterior location along the length of a given blood vessel. They further indicate that the profound changes in blood flow magnitude occurring in zebrafish embryos between 24 and 72 hpf, both in the vein and artery, differentially affect the morphology of arterial and venous ECs [34]. In the artery, increases in vessel diameters and EC sizes correlate, as larger arterial segments are made up of bigger ECs (Fig. 1h, i, Suppl. Table 1). By contrast, ECs in the larger trunk vein do not significantly change in area over time, while those in the smaller diameter tail vein increase in area, albeit to a lesser degree than arterial ECs. This leads to a reverse relationship between vessel diameter and EC sizes:larger diameter vein segments in the trunk contain smaller ECs when compared to smaller diameter tail vein segments (Fig. 1h, i, Suppl. Table 1).

### Changes in blood flow influence arterial diameters and EC sizes while veins respond less

**Fig. 2:**
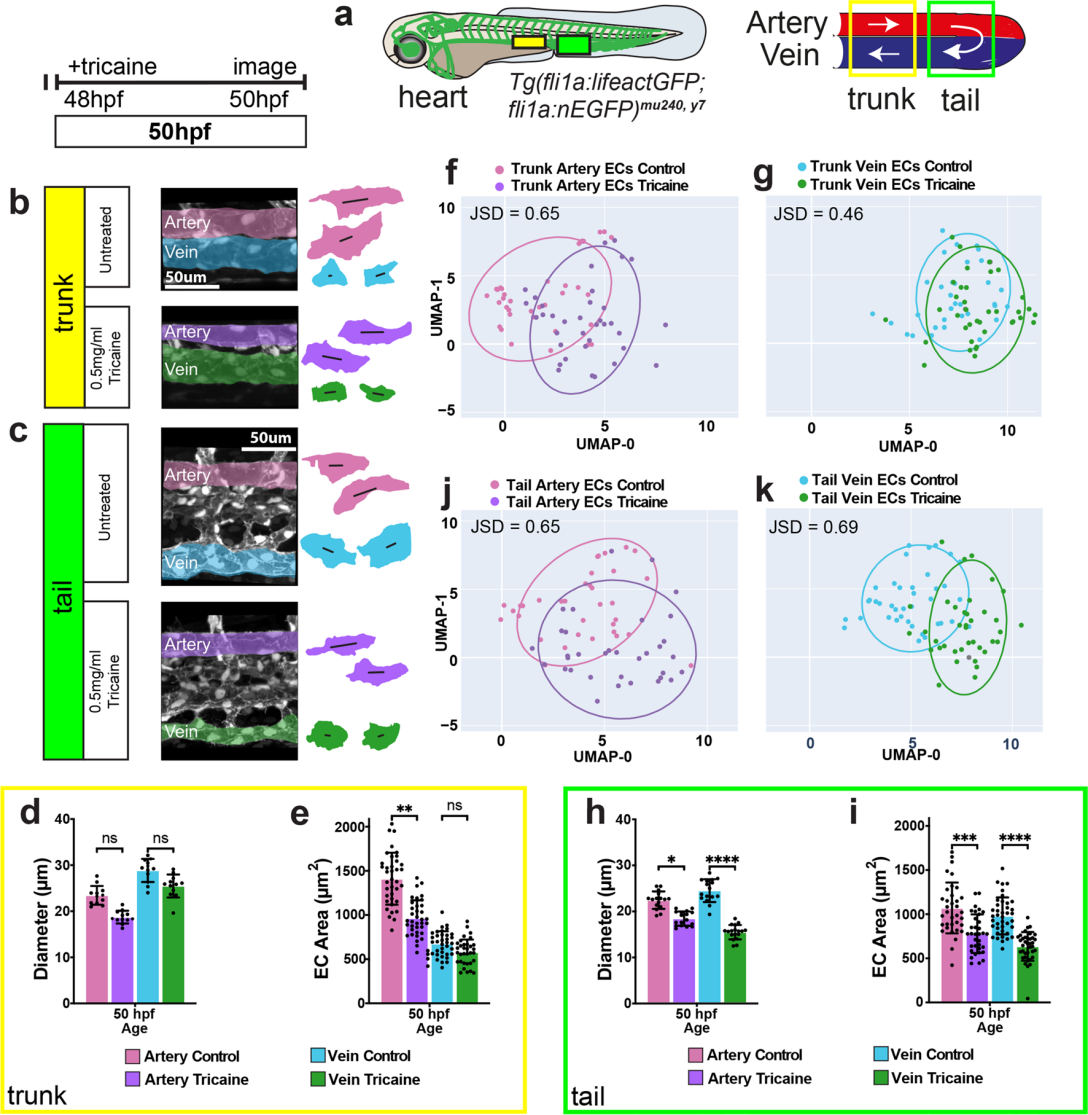
Reducing blood flow affects arterial EC growth, while venous ECs are differentially affected. **a** Schematic of a wildtype zebrafish embryo with the vasculature highlighted in green. The outlined yellow and green boxes indicate the trunk and tail regions, respectively, with magnified views on the right. White arrows represent blood flow. **b, c** Confocal images of the artery and vein in the trunk (**b**) and tail (**c**) regions of wildtype embryos at 50 hpf either untreated or treated with 0.5 mg/ml tricaine for 2 h. **d, e, h, i** Quantifications of vessel diameter and EC area of the artery and vein in the trunk (d, e) and tail (**h, i**) regions. Data analysed by *t*-test. n.s, not significant; *P < 0.05, **P < 0.01, ***P < 0.001, ****P < 0.0001; error bars indicate s.d. *n* = 30–37 cells from trunk artery, 35 cells from trunk vein, 28–34 cells from tail artery, 37–40 cells from tail vein; 26–38 cells from trunk artery, 26–37 cells from trunk vein, 22–34 cells from tail artery, 28–56 cells from tail vein, from 3 untreated or tricaine treated embryos at 24, 36 and 48 hpf each. **f, g, j, k** UMAP plots of arterial and venous cells at 50 hpf in the trunk (**f, g**) and tail (**j, k**) regions. JSD-Jensen Shannon Divergence

To directly test the hypothesis that blood vessels change their diameters according to the shear stress set point model, we reduced blood flow in zebrafish embryos through tricaine treatment for 2 h at 48 hpf and measured blood vessel diameters and EC shapes (Fig. 2a). We chose the 48 hpf time point, as at this time point blood flow dependent angiogenesis of the tail vein is completed [37–39]. We observed that in tricaine-treated embryos, trunk arterial ECs showed a significant reduction in their size in correlation with a decrease in vessel diameter (Fig. 2b, d–f). By contrast, a reduction in flow affected vein diameters and EC sizes to a lesser extent, (Fig. 2b, d, e, g). Thus, in the trunk, blood flow reduction results in the predicted inward remodeling mainly in arteries.

In the tail, both artery [37, 38] and vein EC sizes became smaller, together with a reduction in vessel diameter after tricaine treatment (Fig. 2c, h–k). In summary, our results indicate that arterial diameters respond to changes in blood flow in both trunk and tail regions in a manner that is consistent with the presence of a shear stress set point. We further show that this response is driven through changes in EC shapes, establishing hemodynamic cues as an important determinant of arterial EC shapes. The vein’s response is context-dependent; the trunk vein is much less affected by either increases or decreases in flow and thus does not seem to have a shear stress set point, while tail vein ECs behave like arterial ECs.

### Global loss of endoglin increases EC size in both the artery and vein

**Fig. 3:**
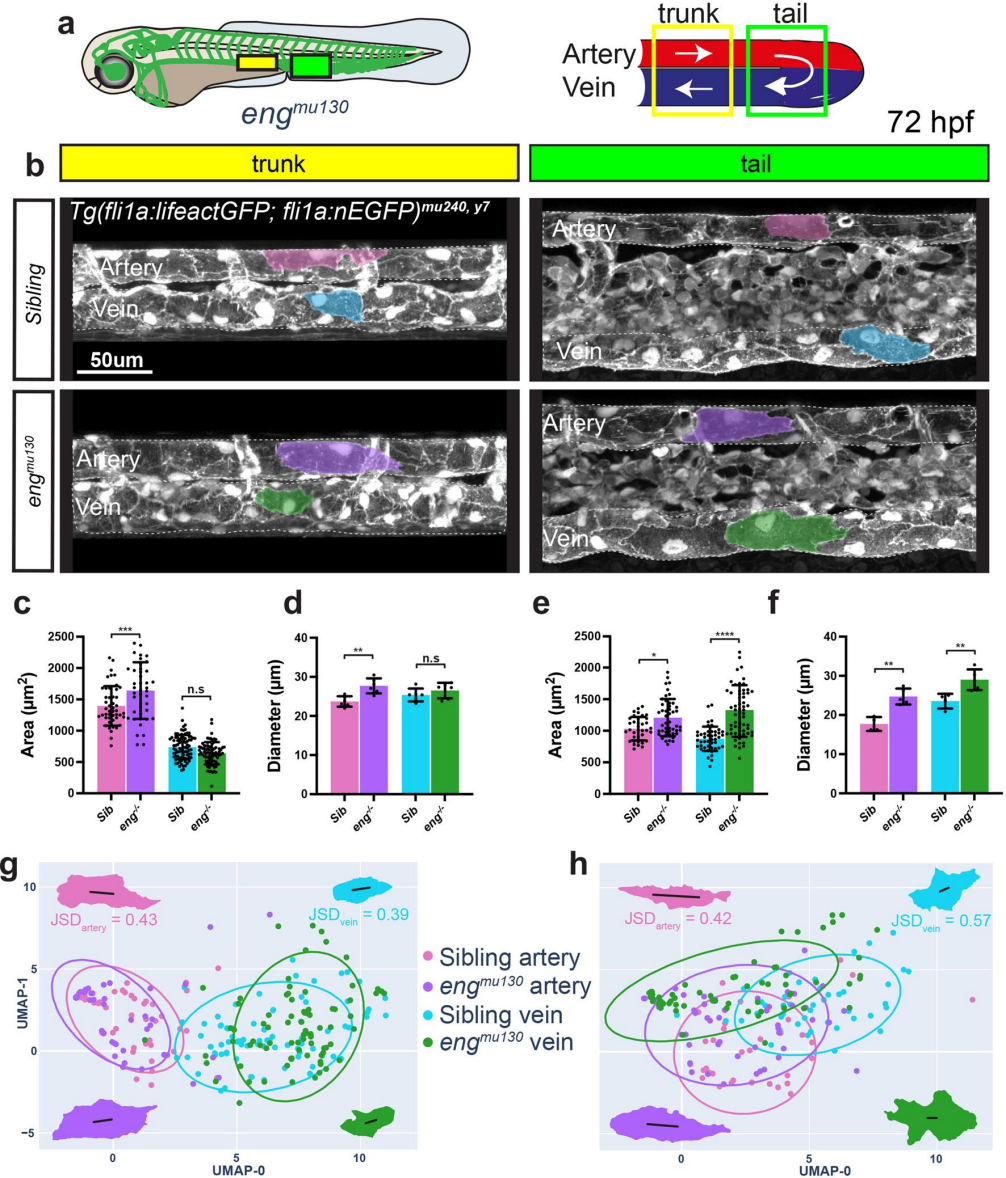
Global loss of Endoglin increases vessel diameters and EC sizes in the dorsal aorta and the posterior cardinal vein. **a** Schematic of a wildtype zebrafish embryo with the vasculature highlighted in green. The outlined yellow and green boxes indicate the trunk and tail regions, respectively, with magnified views on the right. White arrows represent blood flow. **b** Confocal images of the trunk and tail artery and vein at 72 hpf in *eng*^*mu130*^ mutants and siblings. Representative ECs outlined to show differences in cell morphology. **c–f** Quantifications of EC surface areas and vessel diameters in the trunk (**c, d**) and tail (**e, f**) of *eng*^*mu130*^ and sibling embryos at 72 hpf. Data analysed by one-way ANOVA. n.s, not significant; *P < 0.05, **P < 0.01, ***P < 0.001 ****P < 0.0001; error bars indicate s.d. n = 47 cells from 6 siblings, and 42 cells from 6 mutants (trunk artery); 83 cells from 6 siblings, and 80 cells from 6 mutants (trunk vein); 36 cells from 4 siblings, and 47 cells from 5 mutants (tail artery); 45 cells from 4 siblings, and 61 cells from 5 mutants (tail vein). **g, h** UMAP plots of arterial and venous cells at 72 hpf in the trunk (g) and tail (h) regions. JSD-Jensen Shannon Divergence

The finding that veins respond less to changes in blood flow suggests that there are mechanisms operating in veins that spatially restrict their increases in diameter and EC areas in response to flow. We previously showed that the BMP signaling pathway co-receptor Endoglin regulates trunk artery diameters during zebrafish development by controlling EC shapes and sizes [20]. As Endoglin is principally expressed in the vein (Suppl. Figure 2a), we extended our analysis to venous regions of the mutant vasculature (Fig. 3a, b). We found no significant differences in the number, elongation or angle of alignment of *eng* mutant ECs in either the trunk (Suppl. Figure 3a–c) or the tail vessels (Suppl. Figure 3d–f). As shown earlier [20], we observed an increase in EC sizes in trunk arterial ECs, while trunk vein cells did not change in size (Fig. 3c). In the tail, both artery and vein cells increased in area (Fig. 3e). These changes in EC sizes correlated with an increase in the diameters of the respective vessels (Fig. 3d, f). Of note, while we observed a similar percentage of area increase (17%) in both trunk and tail arterial ECs in *eng* mutants compared to siblings, tail vein ECs were 50% larger in mutants compared to siblings (Fig. 3e). This difference is also reflected when using UMAPs to plot EC shape parameters (Fig. 3g, h). Collectively, these observations indicate that although global loss of *eng* affects both arterial and venous diameters mainly through an increase in EC areas, the most prominent effect on EC sizes and shapes can be observed in the tail vein (Suppl. Figure 3 g, h).

### Endoglin and Alk1 function cell autonomously to restrict venous EC size

**Fig. 4:**
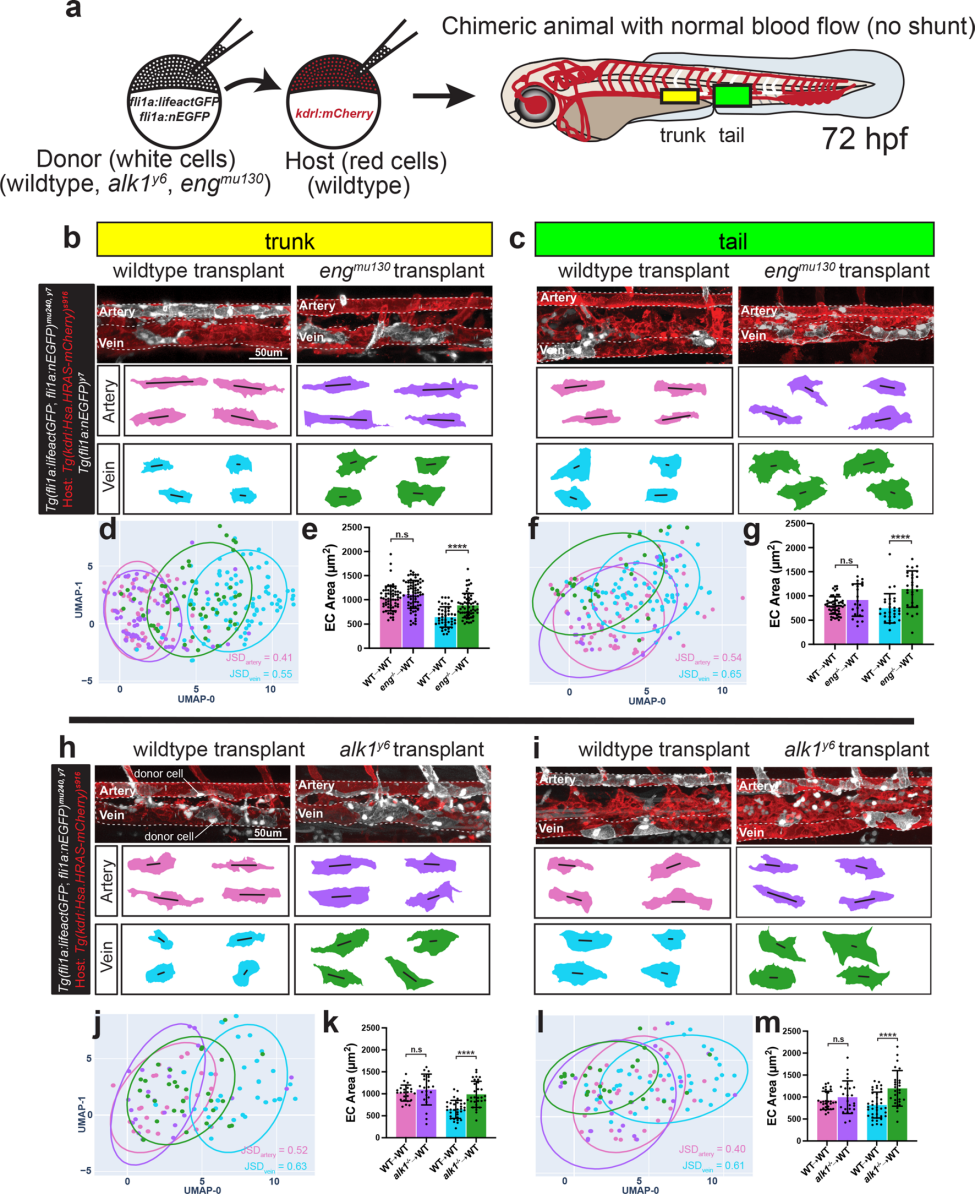
Endoglin and Alk1 regulate venous EC size in a cell autonomous manner. **a** Schematic of transplantation of wildtype, *eng*^*mu130*^ or *alk1*^*y6*^ mutant ECs into a wildtype host. Right hand side shows a 72 hpf mosaic host zebrafish embryo with the vasculature highlighted in red and transplanted ECs in white. The outlined solid boxes indicate the trunk and tail regions analysed. **b, c** Confocal images of the trunk (**b**) and tail (**c**) artery and vein in 72 hpf mosaic embryos transplanted with wildtype or *eng*^*mu130*^ mutant ECs. 2D cell outlines shown below z-stacks. **d–g** UMAP plots and quantifications of EC surface area of transplanted ECs in the trunk (**d, e**) and tail (**f, g**) vessels of the mosaic embryo. JSD-Jensen Shannon Divergence. Data analysed by one-way ANOVA. n.s, not significant; ****P < 0.0001; error bars indicate s.d. *n* = 61 cells transplanted from 11 wildtype, 70 cells transplanted from 13 mutants (trunk artery); 43 cells transplanted from 8 wildtype, 57 cells transplanted from 6 mutants (trunk vein); 54 cells transplanted from 6 wildtype, 22 cells transplanted from 5 mutants (tail artery); 28 cells transplanted from 6 wildtype, 26 cells transplanted from 8 mutants (tail vein). **h, i** Confocal images of the trunk (**h**) and tail (**i**) artery and vein in 72 hpf chimeric embryos transplanted with wildtype or *alk1*^*y6*^ mutant ECs. 2D cell outlines shown below z-stacks. **j–m** UMAP plots and quantifications of EC surface area of transplanted ECs in the trunk (**j, k**) and tail (**l, m**) vessels of the mosaic embryo. JSD-Jensen Shannon Divergence. Data analysed by one-way ANOVA. n.s, not significant; ****P < 0.0001; error bars indicate s.d. *n* = 24 cells transplanted from 5 wildtype, 22 cells transplanted from 4 mutants (trunk artery); 33 cells transplanted from 5 wildtype, 30 cells transplanted from 5 mutants (trunk vein); 30 cells transplanted from 5 wildtype, 25 cells transplanted from 5 mutants (tail artery); 39 cells transplanted from 5 wildtype, 26 cells transplanted from 7 mutants (tail vein)

We previously showed that arterial blood flow is higher in *eng* mutants [20]. As shown in Fig. 1, wildtype arterial ECs increased in size in response to increases in blood flow. This led us to hypothesize that the increase in arterial EC sizes in *eng* mutants might be a secondary effect of increased blood flow, driven by a primary cellular defect in *eng* mutant vein ECs. To address this question, we generated chimeric embryos, in which only a few ECs were mutant in an otherwise wildtype vasculature. We transplanted cells from *eng* mutant or wildtype donor embryos into wildtype recipients and determined the morphology of donor ECs in chimeric embryos at 72 hpf (Fig. 4a). We observed that transplanted mutant arterial ECs were not significantly different in size compared to wildtype arterial ECs. By contrast, transplanted mutant venous ECs were significantly larger than their wildtype counterparts in both the trunk and the tail (Fig. 4b–g). No difference was observed in elongation and angle of alignment between mutant and wildtype transplanted ECs (Suppl. Figure 4a, b). This indicates that expression of *eng* in veins is cell-autonomously required to restrict venous EC sizes and that the arterial EC size increase in *eng* mutants is most likely a secondary response caused by increased flow.

Next, we interrogated whether *alk1*, a gene mainly expressed in arterial ECs (Suppl. Figure 2b), was similarly required to restrict vein EC sizes. The presence of a large shunt in the head, diverting flow from the trunk, precludes an analysis of DA and PCV diameters and EC shapes in global *alk1* mutants [19]. Transplanted *alk1* mutant ECs behaved similarly to *eng* mutant transplanted ECs (Fig. 4h–m). Mutant arterial ECs were not significantly different in size compared to wildtype cells, while mutant venous ECs were nearly 50% larger in both the trunk and the tail (Fig. 4h–m). Elongation and angle of alignment were comparable between mutant and wildtype ECs (Suppl. Figure 4c, d). Together, these findings indicate that the primary cellular defect in both *eng* and *alk1* mutants is in venous ECs, causing a flow-dependent enlargement of these cells.

### Initiation of vessel dilation in endoglin mutants occurs in the tail vein

**Fig. 5:**
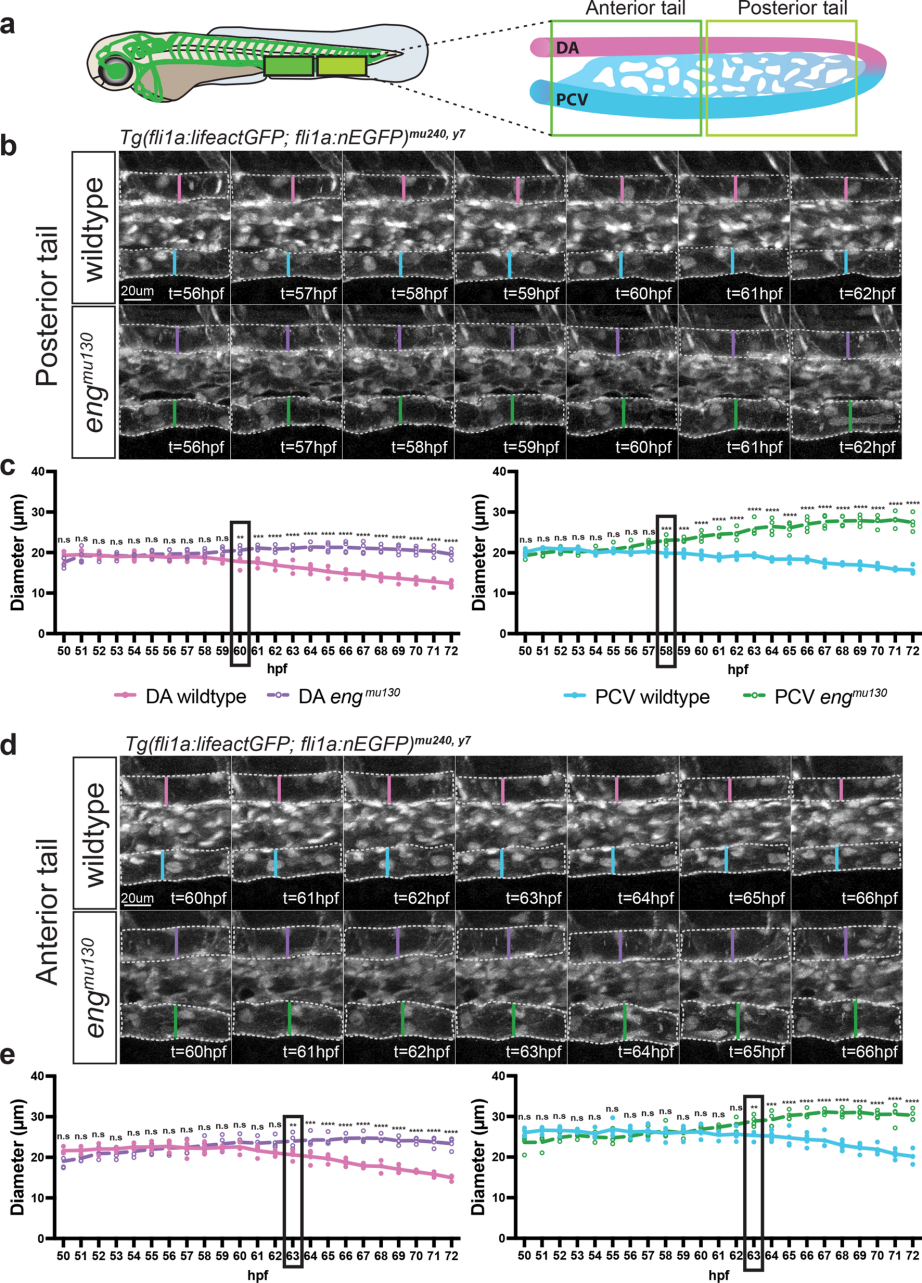
Initiation of vessel dilation in *eng*^*mu130*^ mutants occurs in the tail vein. **a** Schematic of a 72 hpf zebrafish embryo with the vasculature highlighted in green. The outlined solid boxes indicate anterior and posterior sections of the tail vasculature with magnified views on the right. **b, d** Timelapse images of wildtype and *eng*^*mu130*^ mutant artery and vein in the posterior (**b**) and anterior (**d**) tail regions. Vertical lines indicate diameters of artery and vein. **c, e** Quantifications of artery and vein diameters in the posterior (**c**) and anterior (**e**) tail region of wildtype and *eng*^*mu130*^ mutants. Diameters quantified per hour from 50 to 72 hpf timelapse movies. Black outlined boxes represent the timepoints where *eng*^mu130^ mutant and wildtype diameters begin to diverge significantly. Data analysed across genotypes by two-way ANOVA. n.s, not significant; *P < 0.05, **P < 0.01, ***P < 0.001, ****P < 0.0001; error bars indicate s.d. *n* = 4 movies

We reasoned that if the primary defect in *eng* mutants is indeed in vein ECs, we should observe increases in vein diameters prior to increases in artery diameters. To address this question, we performed a time resolved analysis of tail arterial and venous diameters in wildtype and *eng* mutant embryos from 50 to 72 hpf. To accommodate for differences in vessel diameter between anterior and posterior tail regions, we divided the tail into an anterior and a posterior segment (Fig. 5a). We observed that while wildtype vessels decreased their diameters during this time interval, *eng* mutant arteries and veins dilated (Movies 1–4). We noticed a larger increase in *eng* mutant venous compared to arterial diameters in both tail segments. We also observed a difference in the timepoint when mutant diameters diverged from wildtype for the artery versus the vein. The first increase in *eng* mutant vessel diameter was initiated in the posterior tail vein at around 58 hpf, followed by increases in the posterior tail artery diameter at around 60 hpf (Fig. 5b, c; Suppl. Figure 5). At around 63 hpf, the mutant anterior tail artery and vein diverged from wildtype (Fig. 5d, e; Suppl. Figure 5). These data support our hypothesis that in *eng* mutants vein ECs cell autonomously enlarge in response to flow, decreasing flow resistance in the tail vein. This leads to higher flow in arterial vessels, which secondarily enlarge in response to increased flow (Suppl. Figure 6) [41–49].

## Discussion

Despite the importance of maintaining vascular hierarchy, we still do not understand the cellular and molecular mechanisms that spatiotemporally establish blood vessel diameters. Our current study shows profound differences in size and shape of arterial and venous ECs and how these differences affect blood vessel diameters. These findings add to a growing body of whole organism single cell morphology and gene expression data, as performed for the nereid *Platynereis dumerilii* [50]. In the future, this work will allow us to understand how cell morphologies and their underlying gene expression patterns inform organ development and function. Our cell morphology data is limited, as it only contains 2D cell shape information. However, it benefits from being acquired in living animals, avoiding fixation artifacts that can compromise the topologies of tubular organs [51, 52].

Our analysis revealed a positive correlation of EC sizes with vessel diameters for arteries, while we discovered an inverse correlation between cell sizes and vessel diameters for veins. We also uncovered that vein ECs are smaller when compared to arterial ECs and that veins contain more cells in comparison to arteries. At present, we can only speculate as to why this might be the case. Arterial ECs can differentiate through sprouting angiogenesis from venous endothelium, a process that requires downregulation of vein EC proliferation [28, 53–57]. In other settings, slowly cycling precursor cells are also smaller than their differentiated progeny [58]. Thus, vein ECs might be considered the vascular “stem cell pool” from which other vascular lineages differentiate. This view would be consistent with reports showing that the majority of lymphatic ECs are also vein-derived [59].

Increases in EC sizes have been described in several HHT models, including zebrafish and mice, suggesting functional conservation of HHT-causing genes in EC biology [21, 41, 60, 61]. Previous studies deleting SMAD4 in all ECs reported increases in both arterial and venous EC sizes [41, 60], while a recent report analysing *alk1* mutant ECs in either veins or arteries in mouse retinae found increases in cell volumes only in venous ECs [61], corroborating our findings in zebrafish. Together, these results suggest that both in the zebrafish DA and PCV as well as in mouse retinae, increases in arterial EC sizes are a secondary result of aberrant arterial flow patterns.

Recent results show that in larger cells, cell cycle inhibitors are more dilute when compared to cell cycle activators [62, 63]. This could indicate that in Alk1/Endoglin mutants, aberrant increases in cell size subsequently lead to an increase in proliferation and thereby secondarily to an increase in cell numbers in affected vessels, causing even greater increases in blood vessel diameters. Indeed, similar to findings in mammalian HHT models [16, 64], we have observed an increase in vein EC numbers within the fin vasculature of adult *eng* mutants [20]. Jin et al. showed that in AVMs, both *eng* mutant and wildtype cells proliferated more, suggesting that increases in EC proliferation might be a secondary effect due to aberrant EC sizes. Together, these findings illustrate the significance of understanding and separating primary from secondary effects, which are likely occurring due to changes in hemodynamics that result from loss of Alk1/Endoglin function.

We find that increases in blood flow led to an increase in arterial EC sizes together with an increase in artery diameter. Thus, arterial vessels behave according to the shear stress set point theory with diameter changes driven through changes in EC shapes at developmental stages prior to mural cell investment. By contrast, vein ECs react less to increases in flow and maintain their sizes, preventing the uncontrolled diameter increase of a short connection between an artery and a vein, as previously proposed [7]. This suggests a mechanism in venous ECs that tunes activation of a signalling pathway according to the amount of shear stress a given cell encounters. We propose that this pathway acts through Alk1 and Endoglin. Previous work showed that Endoglin can potentiate Alk1 signalling in response to shear stress [5]. SMAD1 nuclear translocation also increased with increases in shear stress up to a set-point value, while decreasing again at higher shear rates [65]. These studies were performed in HUVECs (Human Umbilical Vein Endothelial Cells), and it would be of interest to determine whether increases in shear stress during embryonic development would lead to similar increases in SMAD1 nuclear translocation in vein ECs and presumably BMP signalling pathway activation.

The finding that arterial ECs lacking Alk1 or Endoglin do not show a phenotype when in a normal hemodynamic environment is surprising. However, previous studies reported that small telangiectasias in HHT patients preferentially affected postcapillary venules [31]. Similarly, inducing angiogenesis in *Eng* mutant mice resulted in venomegaly [16]. Specifically deleting *Eng* or *Alk1* in capillary and venous ECs resulted in similar AVM frequency in the mouse retina when compared to pan-endothelial deletion of either gene [29, 30] or when examining shunts in a model of oxygen induced retinopathy [61]. These results suggest that the primary EC cell type affected by loss of Alk1/Endoglin signalling are indeed venous ECs. A notable exception is the brain, where arterial ECs forming the Circle of Willis in zebrafish are most prominently affected [19]. Thus, whether an EC population is affected in mutant animals might also depend on additional parameters, such as angiogenic status.

Earlier studies have linked AVM development to an upregulation of the Phosphatidylinositol 3-kinase (PI3K) pathway [24, 32, 66]. Of note, overactivation of PI3K signalling only leads to vascular malformations in veins and capillaries but not in arteries [67–69]. Thus, differentiated arterial ECs appear to be refractory to both loss of Alk1/Endoglin signalling and enhanced PI3K signalling. In addition to causing vascular malformations, overactivation of PI3K signalling is a hallmark of cancer [70]. Thus, identifying the mechanism through which arterial cells fail to respond to increases in PI3K signalling might also help in developing cancer treatments.

How PI3K signalling might affect EC sizes is currently unknown. Previous studies showed that cell autonomous loss of PI3K signalling led to an increase in EC areas in the mouse retina [71]. However, in Alk1/Endoglin and SMAD4 mutants, increases in EC sizes correlate with an increase in PI3K signalling, and blocking PI3K signalling can rescue vascular phenotypes in Alk1/Endoglin and SMAD4 mutants [21, 32, 66, 72, 73]. It is well established that mTOR downstream of PI3K signalling promotes cell growth through controlling translation and ribosomal biogenesis [74]. Blocking mTOR signalling can reverse shunts in mice lacking BMP9/10 and in Endoglin mutant zebrafish [73, 75], like vascular malformations in mice with overactivated PI3 kinase signalling [38, 67, 76] or in a model of oxygen induced retinopathy [61].

Our findings in chimeric animals help in understanding the puzzling finding that in many vascular lesions in HHT patients only a small number of ECs are mutant [77–79, 83]. Our results now suggest that only a few vein cells need to be mutant and that arterial ECs subsequently respond to higher flow with increases in their areas, as they would in wildtype animals. Thus, a normal physiological response that is necessary to maintain the shear stress set point in arteries results in a maladaptation of these vessels in a disease setting. This response would then lead the arterial vessel, even though it consists of genotypically wildtype cells, to enlarge and become part of an arterio-venous malformation. Further insights into these feedback loops might therefore aid our understanding of the heterogeneity of phenotypes observed in HHT patients and pave the way for better treatment options.

## Methods

### Zebrafish husbandry and strains

All research protocols involving zebrafish were approved by the University of Pennsylvania Institutional Animal Care and Use Committee (Number 806819). Our investigations have (i) local approval and (ii) all procedures conform to the guidelines from the NIH Guide for the Care and Use of Laboratory Animals. Euthanasia was performed using rapid chilling. Zebrafish veterinary care was performed under the supervision of the University Laboratory Animal Resources at the University of Pennsylvania. Zebrafish embryos were maintained in 1 × E3 medium under recommended animal husbandry conditions [80] and in accordance with institutional and national regulatory standards. Transgenic lines and mutants used were *Tg(fli1a:lifeactGFP)*^*mu*240^, *Tg(fli1a:nEGFP)*^*y*7^, *Tg(kdrl:EGFP)*^*s843*^, *Tg(kdrl:Hsa.HRAS-mCherry)*^*s916*^, *eng*^*mu130*^, and *alk1*^*y6*^. References for all zebrafish lines can be obtained on http://zfin.org.

### Live imaging and confocal microscopy

Twenty four to 72 hpf embryos were mounted on a glass-bottom dish using 1% low-melting-point agarose containing 168 mg/l tricaine and 0.003% phenylthiourea (to prevent pigment formation). For time-lapse imaging, a heated microscope stage was used to maintain a constant temperature of 28.5 °C. Confocal *z*-stacks were acquired on an SP8-inverted (Leica Microsystems) or LSM880 (Zeiss) scanning confocal microscope.

### Tricaine treatments

Embryos were dechorionated and treated with 0.5 mg/ml tricaine (Millipore Sigma) in 1 × E3 containing 0.003% phenylthiourea for 2 h intervals at 24, 36, 48 and 72 hpf, and imaged immediately after. This treatment led to a reduction in blood flow without fully stopping flow [81].

### Image processing

Imaris software (Oxford Instruments) was used for image analysis, including maximum intensity projections, mp4 movies, cell shape tracings, and measurement of vessel diameters, cell numbers and cell proliferation. Adobe Illustrator software was used to assemble figures and create schematics.

### Vessel diameter, cell number and cell shape analysis

To calculate vessel diameters, measurements were taken at the midway point between intersegmental vessels (ISVs) no. 7–15 for trunk and ISVs no. 16–24 for tail. The mean was used as an average diameter per embryo per trunk or tail region. Nuclei counts were obtained for 430 µm length of each vessel between ISVs no. 7–15 for trunk and ISVs no. 16–24 for tail.

Cell shape analysis was performed as described previously [20]. Briefly, *Tg(fli1a:lifeactGFP; fli1a:nEGFP)*^*mu*240,*y*7^ double transgenic embryos were imaged with a 40 × air or 63 × water objective. Imaris software was used to manually trace cell outlines with an average of 100 measurement points per cell. Using a custom MATLAB script, these points’ coordinates were first translated and rotated based on the vessel centerline’s vector so that the vessel is positioned on the *x*-axis, and all the cell boundaries’ points are surrounding the *x*-axis. Then the outline coordinates for each cell in their *y* and *z* dimension are fitted in an ellipse or a hyperbola as the cross section of the vessel. Next, their *y* and *z* dimension are flattened into 1 dimension along the fitted curve. Together with their coordinates in *x* dimension, the cell is now projected into a 2-dimensional surface. Next, we fitted the projected cell boundary coordinates in 2D onto an ellipse whose lengths of major axis and minor axis are $a$ and $b$, the center point’s coordinate is ${X}_{c}$, and the angle between the major axis and *x*-axis is $\varphi$. EC elongation is defined as $\frac{a}{b}$. The angle between the EC and the main axis of the vessel is $\varphi$ because all coordinates in 3D are normalized, so the vessel’s main axis is* x*-axis. Instead of EC alignment, which is defined as a combination of angle of alignment and cell elongation, we used the angle of alignment in our cell shape UMAP projection.

### UMAP plots on cell shape parameters

Four cell shape parameters (area, angle, elongation, and perimeter) of each unrolled EC were quantified and exported from a Matlab script into a csv file. We designed a Python script to organize these parameters and map them into UMAPs. Briefly, the four parameters together with their corresponding cells’ identities such as cell name, cell location, vessel type, and age, etc. were organized into a DataFrame in Python. We applied normalization to the four parameters’ dimensions, followed by UMAP projection. We display the first and second dimensions of the projected UMAP space as scatter plots. To show the clustering and distance features of each cell group, we fitted an ellipse to outline the main part of the scattered points of each cell type. Finally, the cell points and the bounding ellipses from the selected cell types were shown on the UMAP plots to compare their similarities and distances. To statistically measure the similarity between cell types, we calculated the Jensen-Shannon Divergence (JSD) between the cell shape parameters’ distribution in their original 4 dimensions, using “gaussian_kde” and “jensenshannon” functions in the Python “scipy” package.

### Proliferation rate

Time-lapse movies were analyzed for cells that displayed nuclear dissociation and subsequent cell division. Proliferation events were counted for each vessel between 24–36, 36–48 and 48–72 hpf and normalized to the total cell number (per 430 µm vessel length) to obtain rates of proliferation.

### Blastomere transplantations

Cell transplantations were performed as described previously [82]. Wildtype, *eng*^*mu130*^ or *alk1*^*y6*^ mutant embryos with *Tg(fli1a:lifeactGFP; fli1a:nEGFP)*^*mu*240,*y*7^ were used as donors, while recipients were wildtype *Tg(kdrl:Hsa.HRAS-mCherry; fli1a:nEGFP)*^*s916,y7*^ embryos.

### In situ hybridization

In situ hybridization was carried out as previously described [20]. Primer sequences to amplify *alk1* were kindly provided by Beth Roman’s lab (University of Pittsburgh).

### Statistical analysis

All data were analyzed using GraphPad Prism or R packages. Graphs were plotted with mean standard deviation (s.d.). *P* < 0.05 was considered statistically significant.

## Supplementary Information

Below is the link to the electronic supplementary material.

Supplementary file1 (AVI 14069 KB)

Supplementary file2 (AVI 13516 KB)

Supplementary file3 (AVI 11235 KB)

Supplementary file4 (AVI 11238 KB)

Supplementary file5 (XLSX 13 KB)

## Data Availability

All code used in this study is available from the authors upon request.

## References

[1] Camelo C, Luschnig S (2021) Cells into tubes: molecular and physical principles underlying lumen formation in tubular organs. Curr Top Dev Biol 143:37–74. 10.1016/bs.ctdb.2020.09.002 33820625 10.1016/bs.ctdb.2020.09.002

[2] Queisser A, Seront E, Boon LM, Vikkula M (2021) Genetic basis and therapies for vascular anomalies. Circ Res 129:155–173. 10.1161/CIRCRESAHA.121.318145 34166070 10.1161/CIRCRESAHA.121.318145

[3] Langille BL (1996) Arterial remodeling: relation to hemodynamics. Can J Physiol Pharmacol 74:834–841 8946070

[4] Tuttle JL, Nachreiner RD, Bhuller AS, Condict KW, Connors BA, Herring BP, Dalsing MC, Unthank JL (2001) Shear level influences resistance artery remodeling: wall dimensions, cell density, and eNOS expression. Am J Physiol Heart Circulat Physiol 281:H1380–1389 10.1152/ajpheart.2001.281.3.H1380 11514310

[5] Baeyens N, Larrivee B, Ola R, Hayward-Piatkowskyi B, Dubrac A, Huang B, Ross TD, Coon BG, Min E, Tsarfati M, Tong H, Eichmann A, Schwartz MA (2016) Defective fluid shear stress mechanotransduction mediates hereditary hemorrhagic telangiectasia. J Cell Biol 214:807–816. 10.1083/jcb.201603106 27646277 10.1083/jcb.201603106 PMC5037412

[6] Rodbard S (1975) Vascular caliber. Cardiology 60:4–49 126799 10.1159/000169701

[7] Pries AR, Hopfner M, le Noble F, Dewhirst MW, Secomb TW (2010) The shunt problem: control of functional shunting in normal and tumour vasculature. Nat Rev Cancer 10:587–593. 10.1038/nrc2895 20631803 10.1038/nrc2895 PMC3109666

[8] Siekmann AF (2023) Biology of vascular mural cells. Development *150*. 10.1242/dev.200271. 10.1242/dev.200271 PMC10482013 37642459

[9] Ando K, Ishii T, Fukuhara S (2021) Zebrafish vascular mural cell biology: recent advances, development, and functions. Life (Basel) 11. 10.3390/life11101041. 10.3390/life11101041 PMC8537713 34685412

[10] Tsuji-Tamura K, Ogawa M (2018) Morphology regulation in vascular endothelial cells. Inflamm Regen 38:25. 10.1186/s41232-018-0083-8 30214642 10.1186/s41232-018-0083-8 PMC6130072

[11] Vanlandewijck M, He L, Mae MA, Andrae J, Ando K, Del Gaudio F, Nahar K, Lebouvier T, Lavina B, Gouveia L, Sun Y, Raschperger E, Rasanen M, Zarb Y, Mochizuki N, Keller A, Lendahl U, Betsholtz C (2018) A molecular atlas of cell types and zonation in the brain vasculature. Nature 554:475–480. 10.1038/nature25739 29443965 10.1038/nature25739

[12] Campinho P, Vilfan A, Vermot J (2020) Blood flow forces in shaping the vascular system: a focus on endothelial cell behavior. Front Physiol 11:552. 10.3389/fphys.2020.00552 32581842 10.3389/fphys.2020.00552 PMC7291788

[13] Gifre-Renom L, Jones EAV (2021) Vessel enlargement in development and pathophysiology. Front Physiol 12:639645. 10.3389/fphys.2021.639645 33716786 10.3389/fphys.2021.639645 PMC7947306

[14] Gallione CJ, Richards JA, Letteboer TG, Rushlow D, Prigoda NL, Leedom TP, Ganguly A, Castells A, Ploos van Amstel JK, Westermann CJ, Pyeritz RE, Marchuk DA (2006) SMAD4 mutations found in unselected HHT patients. J Med Genet 43:793–797. 10.1136/jmg.2006.041517 16613914 10.1136/jmg.2006.041517 PMC2563178

[15] Johnson DW, Berg JN, Baldwin MA, Gallione CJ, Marondel I, Yoon SJ, Stenzel TT, Speer M, Pericak-Vance MA, Diamond A, Guttmacher AE, Jackson CE, Attisano L, Kucherlapati R, Porteous ME, Marchuk DA (1996) Mutations in the activin receptor-like kinase 1 gene in hereditary haemorrhagic telangiectasia type 2. Nat Genet 13:189–195. 10.1038/ng0696-189 8640225 10.1038/ng0696-189

[16] Mahmoud M, Allinson KR, Zhai Z, Oakenfull R, Ghandi P, Adams RH, Fruttiger M, Arthur HM (2010) Pathogenesis of arteriovenous malformations in the absence of endoglin. Circ Res 106:1425–1433. 10.1161/CIRCRESAHA.109.211037 20224041 10.1161/CIRCRESAHA.109.211037

[17] McAllister KA, Grogg KM, Johnson DW, Gallione CJ, Baldwin MA, Jackson CE, Helmbold EA, Markel DS, McKinnon WC, Murrell J et al (1994) Endoglin, a TGF-beta binding protein of endothelial cells, is the gene for hereditary haemorrhagic telangiectasia type 1. Nat Genet 8:345–351. 10.1038/ng1294-345 7894484 10.1038/ng1294-345

[18] Park SO, Wankhede M, Lee YJ, Choi EJ, Fliess N, Choe SW, Oh SH, Walter G, Raizada MK, Sorg BS, Oh SP (2009) Real-time imaging of de novo arteriovenous malformation in a mouse model of hereditary hemorrhagic telangiectasia. J Clin Invest 119:3487–3496. 10.1172/JCI39482 19805914 10.1172/JCI39482 PMC2769195

[19] Roman BL, Pham VN, Lawson ND, Kulik M, Childs S, Lekven AC, Garrity DM, Moon RT, Fishman MC, Lechleider RJ, Weinstein BM (2002) Disruption of acvrl1 increases endothelial cell number in zebrafish cranial vessels. Development 129:3009–3019 12050147 10.1242/dev.129.12.3009

[20] Sugden WW, Meissner R, Aegerter-Wilmsen T, Tsaryk R, Leonard EV, Bussmann J, Hamm MJ, Herzog W, Jin Y, Jakobsson L, Denz C, Siekmann AF (2017) Endoglin controls blood vessel diameter through endothelial cell shape changes in response to haemodynamic cues. Nat Cell Biol 19:653–665. 10.1038/ncb3528 28530658 10.1038/ncb3528 PMC5455977

[21] Banerjee K, Lin Y, Gahn J, Cordero J, Gupta P, Mohamed I, Graupera M, Dobreva G, Schwartz MA, Ola R (2023) SMAD4 maintains the fluid shear stress set point to protect against arterial-venous malformations. J Clin Invest, 133. 10.1172/JCI168352. 10.1172/JCI168352 PMC10503796 37490341

[22] Genet G, Genet N, Paila U, Cain SR, Cwiek A, Chavkin NW, Serbulea V, Figueras A, Cerda P, McDonnell SP, Sankaranarayanan D, Huba M, Nelson EA, Riera-Mestre A, Hirschi KK (2024) Induced endothelial cell cycle arrest prevents arteriovenous malformations in hereditary hemorrhagic telangiectasia. Circulation 149:944–962. 10.1161/CIRCULATIONAHA.122.062952 38126211 10.1161/CIRCULATIONAHA.122.062952 PMC10954087

[23] Dinakaran S, Zhao H, Tang Y, Wang Z, Ruiz S, Nomura-Kitabayashi A, Blanc L, Faughnan ME, Marambaud P (2023) CDK6-mediated endothelial cell cycle acceleration drives arteriovenous malformations in hereditary hemorrhagic telangiectasia. bioRxiv. 10.1101/2023.09.15.554413. 10.1038/s44161-024-00550-9 PMC11651362 39487364

[24] Jin Y, Muhl L, Burmakin M, Wang Y, Duchez AC, Betsholtz C, Arthur HM, Jakobsson L (2017) Endoglin prevents vascular malformation by regulating flow-induced cell migration and specification through VEGFR2 signalling. Nat Cell Biol 19:639–652. 10.1038/ncb3534 28530660 10.1038/ncb3534 PMC5467724

[25] Tual-Chalot S, Mahmoud M, Allinson KR, Redgrave RE, Zhai Z, Oh SP, Fruttiger M, Arthur HM (2014) Endothelial depletion of Acvrl1 in mice leads to arteriovenous malformations associated with reduced endoglin expression. PLoS ONE 9:e98646. 10.1371/journal.pone.0098646 24896812 10.1371/journal.pone.0098646 PMC4045906

[26] Urness LD, Sorensen LK, Li DY (2000) Arteriovenous malformations in mice lacking activin receptor-like kinase-1. Nat Genet 26:328–331. 10.1038/81634 11062473 10.1038/81634

[27] Poduri A, Chang AH, Raftrey B, Rhee S, Van M, Red-Horse K (2017) Endothelial cells respond to the direction of mechanical stimuli through SMAD signaling to regulate coronary artery size. Development 144:3241–3252. 10.1242/dev.150904 28760815 10.1242/dev.150904 PMC5612251

[28] Lee HW, Xu Y, He L, Choi W, Gonzalez D, Jin SW, Simons M (2021) Role of venous endothelial cells in developmental and pathologic angiogenesis. Circulation 144:1308–1322. 10.1161/CIRCULATIONAHA.121.054071 34474596 10.1161/CIRCULATIONAHA.121.054071 PMC9153651

[29] Park H, Furtado J, Poulet M, Chung M, Yun S, Lee S, Sessa WC, Franco CA, Schwartz MA, Eichmann A (2021) Defective flow-migration coupling causes arteriovenous malformations in hereditary hemorrhagic telangiectasia. Circulation 144:805–822. 10.1161/CIRCULATIONAHA.120.053047 34182767 10.1161/CIRCULATIONAHA.120.053047 PMC8429266

[30] Singh E, Redgrave RE, Phillips HM, Arthur HM (2020) Arterial endoglin does not protect against arteriovenous malformations. Angiogenesis 23:559–566. 10.1007/s10456-020-09731-z 32506200 10.1007/s10456-020-09731-z PMC7524831

[31] Braverman IM, Keh A, Jacobson BS (1990) Ultrastructure and three-dimensional organization of the telangiectases of hereditary hemorrhagic telangiectasia. J Invest Dermatol 95:422–427. 10.1111/1523-1747.ep12555569 2212727 10.1111/1523-1747.ep12555569

[32] Ola R, Dubrac A, Han J, Zhang F, Fang JS, Larrivee B, Lee M, Urarte AA, Kraehling JR, Genet G, Hirschi KK, Sessa WC, Canals FV, Graupera M, Yan M, Young LH, Oh PS, Eichmann A (2016) PI3 kinase inhibition improves vascular malformations in mouse models of hereditary haemorrhagic telangiectasia. Nat Commun 7:13650. 10.1038/ncomms13650 27897192 10.1038/ncomms13650 PMC5141347

[33] Maung Ye SS, Kim JK, Carretero NT, Phng LK (2022) High-throughput imaging of blood flow reveals developmental changes in distribution patterns of hemodynamic quantities in developing zebrafish. Front Physiol 13:881929. 10.3389/fphys.2022.881929 35795647 10.3389/fphys.2022.881929 PMC9251365

[34] Santoso F, Sampurna BP, Lai Y-H, Liang S-T, Hao E, Chen J-R, Hsiao C-D (2019) Development of a simple ImageJ-based method for dynamic blood flow tracking in zebrafish embryos and its application in drug toxicity evaluation. Inventions 4:65

[35] Bertrand JY, Chi NC, Santoso B, Teng S, Stainier DY, Traver D (2010) Haematopoietic stem cells derive directly from aortic endothelium during development. Nature 464:108–111. 10.1038/nature08738 20154733 10.1038/nature08738 PMC2858358

[36] Wakayama Y, Fukuhara S, Ando K, Matsuda M, Mochizuki N (2015) Cdc42 mediates Bmp-induced sprouting angiogenesis through Fmnl3-driven assembly of endothelial filopodia in zebrafish. Dev Cell 32:109–122. 10.1016/j.devcel.2014.11.024 25584797 10.1016/j.devcel.2014.11.024

[37] Karthik S, Djukic T, Kim JD, Zuber B, Makanya A, Odriozola A, Hlushchuk R, Filipovic N, Jin SW, Djonov V (2018) Synergistic interaction of sprouting and intussusceptive angiogenesis during zebrafish caudal vein plexus development. Sci Rep 8:9840. 10.1038/s41598-018-27791-6 29959335 10.1038/s41598-018-27791-6 PMC6026200

[38] Kugler E, Snodgrass R, Bowley G, Plant K, Serbanovic-Canic J, Hamilton N, Evans PC, Chico T, Armitage P (2021) The effect of absent blood flow on the zebrafish cerebral and trunk vasculature. Vasc Biol 3:1–16. 10.1530/VB-21-0009 34522840 10.1530/VB-21-0009 PMC8428019

[39] Xie X, Zhou T, Wang Y, Chen H, Lei D, Huang L, Wang Y, Jin X, Sun T, Tan J, Yin T, Huang J, Gregersen H, Wang G (2018) Blood flow regulates zebrafish caudal vein plexus angiogenesis by ERK5-klf2a-nos2b signaling. Curr Mol Med 18:3–14. 10.2174/1566524018666180322153432 29577856 10.2174/1566524018666180322153432

[40] Larson JD, Wadman SA, Chen E, Kerley L, Clark KJ, Eide M, Lippert S, Nasevicius A, Ekker SC, Hackett PB, Essner JJ (2004) Expression of VE-cadherin in zebrafish embryos: a new tool to evaluate vascular development. Dev Dyn 231:204–213 15305301 10.1002/dvdy.20102

[41] Crist AM, Zhou X, Garai J, Lee AR, Thoele J, Ullmer C, Klein C, Zabaleta J, Meadows SM (2019) Angiopoietin-2 inhibition rescues arteriovenous malformation in a Smad4 hereditary hemorrhagic telangiectasia mouse model. Circulation 139:2049–2063. 10.1161/CIRCULATIONAHA.118.036952 30744395 10.1161/CIRCULATIONAHA.118.036952 PMC6478529

[42] Hollnagel A, Oehlmann V, Heymer J, Ruther U, Nordheim A (1999) Id genes are direct targets of bone morphogenetic protein induction in embryonic stem cells. J Biol Chem 274:19838–19845. 10.1074/jbc.274.28.19838 10391928 10.1074/jbc.274.28.19838

[43] Nakao A, Afrakhte M, Moren A, Nakayama T, Christian JL, Heuchel R, Itoh S, Kawabata M, Heldin NE, Heldin CH, ten Dijke P (1997) Identification of Smad7, a TGFbeta-inducible antagonist of TGF-beta signalling. Nature 389:631–635. 10.1038/39369 9335507 10.1038/39369

[44] Wang HU, Chen ZF, Anderson DJ (1998) Molecular distinction and angiogenic interaction between embryonic arteries and veins revealed by ephrin-B2 and its receptor Eph-B4. Cell 93:741–753 9630219 10.1016/s0092-8674(00)81436-1

[45] Fernandez-Chacon M, Garcia-Gonzalez I, Muhleder S, Benedito R (2021) Role of Notch in endothelial biology. Angiogenesis 24:237–250. 10.1007/s10456-021-09793-7 34050878 10.1007/s10456-021-09793-7

[46] Leesch F, Lorenzo-Orts L, Pribitzer C, Grishkovskaya I, Roehsner J, Chugunova A, Matzinger M, Roitinger E, Belacic K, Kandolf S, Lin TY, Mechtler K, Meinhart A, Haselbach D, Pauli A (2023) A molecular network of conserved factors keeps ribosomes dormant in the egg. Nature 613:712–720. 10.1038/s41586-022-05623-y 36653451 10.1038/s41586-022-05623-y PMC7614339

[47] Heumos L, Schaar AC, Lance C, Litinetskaya A, Drost F, Zappia L, Lucken MD, Strobl DC, Henao J, Curion F, Practices S-C, Schiller HB, Theis FJ (2023) Best practices for single-cell analysis across modalities. Nat Rev Genet 24:550–572. 10.1038/s41576-023-00586-w 37002403 10.1038/s41576-023-00586-w PMC10066026

[48] Squair JW, Gautier M, Kathe C, Anderson MA, James ND, Hutson TH, Hudelle R, Qaiser T, Matson KJE, Barraud Q, Levine AJ, La Manno G, Skinnider MA, Courtine G (2021) Confronting false discoveries in single-cell differential expression. Nat Commun 12:5692. 10.1038/s41467-021-25960-2 34584091 10.1038/s41467-021-25960-2 PMC8479118

[49] Ochs RL, Lischwe MA, Spohn WH, Busch H (1985) Fibrillarin: a new protein of the nucleolus identified by autoimmune sera. Biol Cell 54:123–133. 10.1111/j.1768-322x.1985.tb00387.x 2933102 10.1111/j.1768-322x.1985.tb00387.x

[50] Vergara HM, Pape C, Meechan KI, Zinchenko V, Genoud C, Wanner AA, Mutemi KN, Titze B, Templin RM, Bertucci PY, Simakov O, Durichen W, Machado P, Savage EL, Schermelleh L, Schwab Y, Friedrich RW, Kreshuk A, Tischer C, Arendt D (2021) Whole-body integration of gene expression and single-cell morphology. Cell 184(4819–4837):e4822. 10.1016/j.cell.2021.07.017 10.1016/j.cell.2021.07.017 PMC8445025 34380046

[51] Maunsbach AB (1966) The influence of different fixatives and fixation methods on the ultrastructure of rat kidney proximal tubule cells. II. Effects of varying osmolality, ionic strength, buffer system and fixative concentration of glutaraldehyde solutions. J Ultrastruct Res 15:283–309. 10.1016/s0022-5320(66)80110-7 5328615 10.1016/s0022-5320(66)80110-7

[52] Schwarzmaier SM, Knarr MRO, Hu S, Erturk A, Hellal F, Plesnila N (2022) Perfusion pressure determines vascular integrity and histomorphological quality following perfusion fixation of the brain. J Neurosci Methods 372:109493. 10.1016/j.jneumeth.2022.109493 35151669 10.1016/j.jneumeth.2022.109493

[53] Bussmann J, Wolfe SA, Siekmann AF (2011) Arterial-venous network formation during brain vascularization involves hemodynamic regulation of chemokine signaling. Development 138:1717–1726. 10.1242/dev.059881 21429983 10.1242/dev.059881 PMC3074448

[54] Chavkin NW, Genet G, Poulet M, Jeffery ED, Marziano C, Genet N, Vasavada H, Nelson EA, Acharya BR, Kour A, Aragon J, McDonnell SP, Huba M, Sheynkman GM, Walsh K, Hirschi KK (2022) Endothelial cell cycle state determines propensity for arterial-venous fate. Nat Commun 13:5891. 10.1038/s41467-022-33324-7 36202789 10.1038/s41467-022-33324-7 PMC9537338

[55] Fang JS, Coon BG, Gillis N, Chen Z, Qiu J, Chittenden TW, Burt JM, Schwartz MA, Hirschi KK (2017) Shear-induced Notch-Cx37-p27 axis arrests endothelial cell cycle to enable arterial specification. Nat Commun 8:2149. 10.1038/s41467-017-01742-7 29247167 10.1038/s41467-017-01742-7 PMC5732288

[56] Red-Horse K, Siekmann AF (2019) Veins and arteries build hierarchical branching patterns differently: bottom-up versus top-down. BioEssays 41:e1800198. 10.1002/bies.201800198 30805984 10.1002/bies.201800198 PMC6478158

[57] Xu C, Hasan SS, Schmidt I, Rocha SF, Pitulescu ME, Bussmann J, Meyen D, Raz E, Adams RH, Siekmann AF (2014) Arteries are formed by vein-derived endothelial tip cells. Nat Commun 5:5758. 10.1038/ncomms6758 25502622 10.1038/ncomms6758 PMC4275597

[58] Li Q, Rycaj K, Chen X, Tang DG (2015) Cancer stem cells and cell size: a causal link? Semin Cancer Biol 35:191–199. 10.1016/j.semcancer.2015.07.002 26241348 10.1016/j.semcancer.2015.07.002 PMC4651715

[59] Grimm L, Hogan BM (2021) Network patterning, morphogenesis and growth in lymphatic vascular development. Curr Top Dev Biol 143:151–204. 10.1016/bs.ctdb.2020.10.007 33820621 10.1016/bs.ctdb.2020.10.007

[60] Crist AM, Lee AR, Patel NR, Westhoff DE, Meadows SM (2018) Vascular deficiency of Smad4 causes arteriovenous malformations: a mouse model of Hereditary Hemorrhagic Telangiectasia. Angiogenesis 21:363–380. 10.1007/s10456-018-9602-0 29460088 10.1007/s10456-018-9602-0 PMC5878194

[61] Ouarne M, Pena A, Ramalho D, Conchinha N, Costa T, Figueiredo A, Saraiva M, Carvalho Y, Misikova L, Oh P, Franco CA (2023) A non-genetic model of vascular shunts informs on the cellular mechanisms of formation and resolution of arteriovenous malformations. bioRxiv, 2023.2008.2021.554159. 10.1093/cvr/cvae160 PMC11629978 39308243

[62] Chen Y, Zhao G, Zahumensky J, Honey S, Futcher B (2020) Differential scaling of gene expression with cell size may explain size control in budding yeast. Mol Cell 78(359–370):e356. 10.1016/j.molcel.2020.03.012 10.1016/j.molcel.2020.03.012 PMC7963363 32246903

[63] Xie S, Swaffer M, Skotheim JM (2022) Eukaryotic cell size control and its relation to biosynthesis and senescence. Annu Rev Cell Dev Biol 38:291–319. 10.1146/annurev-cellbio-120219-040142 35562854 10.1146/annurev-cellbio-120219-040142

[64] Jin D, Zhu D, Fang Y, Chen Y, Yu G, Pan W, Liu D, Li F, Zhong TP (2017) Vegfa signaling regulates diverse artery/vein formation in vertebrate vasculatures. J Genet Genom 44:483–492. 10.1016/j.jgg.2017.07.005. 10.1016/j.jgg.2017.07.005 29037991

[65] Baeyens N, Nicoli S, Coon BG, Ross TD, Van den Dries K, Han J, Lauridsen HM, Mejean CO, Eichmann A, Thomas JL, Humphrey JD, Schwartz MA (2015) Vascular remodeling is governed by a VEGFR3-dependent fluid shear stress set point. eLife 4. 10.7554/eLife.04645. 10.7554/eLife.04645 PMC4337723 25643397

[66] Alsina-Sanchis E, Garcia-Ibanez Y, Figueiredo AM, Riera-Domingo C, Figueras A, Matias-Guiu X, Casanovas O, Botella LM, Pujana MA, Riera-Mestre A, Graupera M, Vinals F (2018) ALK1 loss results in vascular hyperplasia in mice and humans through PI3K activation. Arterioscler Thromb Vasc Biol 38:1216–1229. 10.1161/ATVBAHA.118.310760 29449337 10.1161/ATVBAHA.118.310760

[67] Castel, P., Carmona, F.J., Grego-Bessa, J., Berger, M.F., Viale, A., Anderson, K.V., Bague, S., Scaltriti, M., Antonescu, C.R., Baselga, E., and Baselga, J. (2016). Somatic PIK3CA mutations as a driver of sporadic venous malformations. Science translational medicine *8*, 332ra342. 10.1126/scitranslmed.aaf1164. 10.1126/scitranslmed.aaf1164 PMC4962922 27030594

[68] Castillo SD, Tzouanacou E, Zaw-Thin M, Berenjeno IM, Parker VE, Chivite I, Mila-Guasch M, Pearce W, Solomon I, Angulo-Urarte A, Figueiredo AM, Dewhurst RE, Knox RG, Clark GR, Scudamore CL, Badar A, Kalber TL, Foster J, Stuckey DJ, David AL, Phillips WA, Lythgoe MF, Wilson V, Semple RK, Sebire NJ, Kinsler VA, Graupera M, Vanhaesebroeck B (2016) Somatic activating mutations in Pik3ca cause sporadic venous malformations in mice and humans. Sci Transl Med 8:332ra343. 10.1126/scitranslmed.aad9982. 10.1126/scitranslmed.aad9982 PMC5973268 27030595

[69] Limaye N, Kangas J, Mendola A, Godfraind C, Schlogel MJ, Helaers R, Eklund L, Boon LM, Vikkula M (2015) Somatic activating PIK3CA mutations cause venous malformation. Am J Hum Genet 97:914–921. 10.1016/j.ajhg.2015.11.011 26637981 10.1016/j.ajhg.2015.11.011 PMC4678782

[70] Goncalves MD, Hopkins BD, Cantley LC (2018) Phosphatidylinositol 3-kinase, growth disorders, and cancer. N Engl J Med 379:2052–2062. 10.1056/NEJMra1704560 30462943 10.1056/NEJMra1704560

[71] Angulo-Urarte A, Casado P, Castillo SD, Kobialka P, Kotini MP, Figueiredo AM, Castel P, Rajeeve V, Mila-Guasch M, Millan J, Wiesner C, Serra H, Muixi L, Casanovas O, Vinals F, Affolter M, Gerhardt H, Huveneers S, Belting HG, Cutillas PR, Graupera M (2018) Endothelial cell rearrangements during vascular patterning require PI3-kinase-mediated inhibition of actomyosin contractility. Nat Commun 9:4826. 10.1038/s41467-018-07172-3 30446640 10.1038/s41467-018-07172-3 PMC6240100

[72] Ola R, Kunzel SH, Zhang F, Genet G, Chakraborty R, Pibouin-Fragner L, Martin K, Sessa W, Dubrac A, Eichmann A (2018) SMAD4 prevents flow induced arteriovenous malformations by inhibiting casein kinase 2. Circulation 138:2379–2394. 10.1161/CIRCULATIONAHA.118.033842 29976569 10.1161/CIRCULATIONAHA.118.033842 PMC6309254

[73] Snodgrass RO, Govindpani K, Plant K, Kugler EC, Doh C, Dawson T, McCormack LE, Arthur HM, Chico TJA (2023) Therapeutic targeting of vascular malformation in a zebrafish model of hereditary haemorrhagic telangiectasia. Dis Model Mech 16. 10.1242/dmm.049567. 10.1242/dmm.049567 PMC10110397 36861761

[74] Wang X, Proud CG (2006) The mTOR pathway in the control of protein synthesis. Physiology (Bethesda) 21:362–369. 10.1152/physiol.00024.2006 16990457 10.1152/physiol.00024.2006

[75] Ruiz S, Zhao H, Chandakkar P, Papoin J, Choi H, Nomura-Kitabayashi A, Patel R, Gillen M, Diao L, Chatterjee PK, He M, Al-Abed Y, Wang P, Metz CN, Oh SP, Blanc L, Campagne F, Marambaud P (2020) Correcting Smad1/5/8, mTOR, and VEGFR2 treats pathology in hereditary hemorrhagic telangiectasia models. J Clin Invest 130:942–957. 10.1172/JCI127425 31689244 10.1172/JCI127425 PMC6994128

[76] di Blasio L, Puliafito A, Gagliardi PA, Comunanza V, Somale D, Chiaverina G, Bussolino F, Primo L (2018) PI3K/mTOR inhibition promotes the regression of experimental vascular malformations driven by PIK3CA-activating mutations. Cell Death Dis 9:45. 10.1038/s41419-017-0064-x 29352118 10.1038/s41419-017-0064-x PMC5833448

[77] Best DH, Vaughn C, McDonald J, Damjanovich K, Runo JR, Chibuk JM, Bayrak-Toydemir P (2011) Mosaic ACVRL1 and ENG mutations in hereditary haemorrhagic telangiectasia patients. J Med Genet 48:358–360. 10.1136/jmg.2010.088286 21378382 10.1136/jmg.2010.088286

[78] Clarke JM, Alikian M, Xiao S, Kasperaviciute D, Thomas E, Turbin I, Olupona K, Cifra E, Curetean E, Ferguson T, Redhead J, Genomics England Research, C., Shovlin CL (2020) Low grade mosaicism in hereditary haemorrhagic telangiectasia identified by bidirectional whole genome sequencing reads through the 100,000 Genomes Project clinical diagnostic pipeline. J Med Genet 57:859–862. 10.1136/jmedgenet-2019-106794 32303606 10.1136/jmedgenet-2019-106794 PMC7691802

[79] Torring PM, Kjeldsen AD, Ousager LB, Brusgaard K (2018) ENG mutational mosaicism in a family with hereditary hemorrhagic telangiectasia. Mol Genet Genomic Med 6:121–125. 10.1002/mgg3.361 29243366 10.1002/mgg3.361 PMC5823686

[80] Westerfield M (1993) The Zebrafish Book. University of Oregon Press, Eugene, OR

[81] Kochhan E, Lenard A, Ellertsdottir E, Herwig L, Affolter M, Belting HG, Siekmann AF (2013) Blood flow changes coincide with cellular rearrangements during blood vessel pruning in zebrafish embryos. PLoS ONE 8:e75060. 10.1371/journal.pone.0075060 24146748 10.1371/journal.pone.0075060 PMC3795766

[82] Siekmann AF, Lawson ND (2007) Notch signalling limits angiogenic cell behaviour in developing zebrafish arteries. Nature 445:781–784. 10.1038/Nature05577 17259972 10.1038/nature05577

[83] Snellings DA, Gallione CJ, Clark DS, Vozoris NT, Faughnan ME, Marchuk DA (2019) Somatic mutations in vascular malformations of hereditary hemorrhagic telangiectasia result in bi-allelic loss of ENG or ACVRL1. Am J Hum Genet 105(5):894–906. 10.1016/j.ajhg.2019.09.010 31630786 10.1016/j.ajhg.2019.09.010 PMC6848992

